# Measures to maintain regular operations and prevent outbreaks of SARS-CoV-2 in childcare facilities or schools under pandemic conditions and co-circulation of other respiratory pathogens

**DOI:** 10.3205/dgkh000357

**Published:** 2020-09-15

**Authors:** Arne Simon, Johannes Huebner, Reinhard Berner, Alasdair P. S. Munro, Martin Exner, Hans-Iko Huppertz, Peter Walger

**Affiliations:** 1 Pediatric Oncology and Hematology, Children’s Hospital Medical Center, University Clinics, Homburg, Germany; 2 Division of Pediatric Infectious Diseases, Dr. von Hauner Children’s Hospital, Munich University Hospital, Munich, Germany; 3 Department of Pediatrics, University Hospital and Medical Faculty Carl Gustav Carus, Technische Universität Dresden, Dresden, Germany; 4 NIHR Southampton Clinical Research Facility, University Hospital Southampton NHS Foundation Trust, Southampton, United Kingdom; 5 NIHR Southampton Biomedical Research Centre, University Hospital Southampton NHS Foundation Trust, Southampton, United Kingdom; 6 Institute of Hygiene and Public Health, University of Bonn, Bonn, Germany; 7 Bremen, Germany; 8 German Society of Hospital Hygiene, Berlin, Germany

## Abstract

After the lockdown and the end of the summer holidays, day-cares and schools need to be reopened and (despite the continued circulation of the new coronavirus SARS-CoV-2) kept open. The need for opening up arises from the right of children to education, participation, support and care. This is possible if appropriate hygiene measures are implemented and community transmission remains stable. In addition, the safety of educators, teachers and carers must be a priority and needs to be addressed by appropriate measures. Finally, the needs of families must also be taken into account. The following document describes in detail how these objectives can be achieved.

## Zusammenfassung

Nach dem Lockdown und dem Ende der großen Ferien sollen die Gemeinschaftseinrichtungen und Schulen für Kinder und Jugendliche wieder geöffnet und (trotz fortbestehender Zirkulation des neuen Coronavirus SARS-CoV-2) offen gehalten werden. Die Notwendigkeit zur Öffnung ergibt sich aus dem Recht der Kinder und Jugendlichen auf Bildung, Teilhabe, Förderung und Betreuung. Dies ist dann möglich, wenn angemessene Hygienemaßnahmen durchgeführt werden. Zudem muss die Sicherheit von Erziehern, Lehrern und Betreuern durch angemessene Maßnahmen gewährleistet werden. Schließlich ist auch den Bedürfnissen der Familien Rechnung zu tragen. Das folgende Dokument beschreibt detailliert, wie diese Ziele erreicht werden können.

## Introduction

Although new findings on this topic have been published continuously since the beginning of the pandemic, not all questions concerning the role of children and adolescents in the pandemic spread of SARS-CoV-2 (the pathogen causing COVID-19) have been conclusively clarified yet. All those involved are called upon to actively participate in scientific studies.

In comparison to adults, children and adolescents have, based on the findings to date

- a lower frequency of infection [1], [2], [3], [4], [5] (some, but by far not all studies were conducted during the lockdown, which is a methodological limitation);
- a lower average severity of disease and associated with this;
- a significantly lower risk of the most serious, including fatal, courses;
- from CoVID-19 [6], [7], [8], [9], [10], [11], [12], [13], [14], [15], [16], [17], [18], [19], [20], [21], [22], [23], [24].

There is also evidence that children and adolescents (at least up to 14 years) [25] transmit SARS-CoV-2 to other people less frequently than adults [26], [27], [28], [29], [30], [31], [32]. The data for children up to the age of 10 seem to indicate that their role for transmission dynamics is less important than for adolescents aged 14 and more [25], [33]. 

The scientific publications since the end of May 2020 have confirmed the recommendations of the statement of several medical societies [34], even though discussions on the effectiveness of individual non-pharmaceutical measures continue [35]. According to the authors' current assessment, teachers in schools and staff in day-care facilities have only a low risk of infection through contact with potentially infected children if basic hygiene measures are adhered to [36], [37]. This risk is not increased compared to the risk of infection through contact with adult SARS-CoV-2-infected people in public or private settings [25].

The currently available data speak in favour of a strategy of future prevention concepts differentiated according to the age of the children and adolescents in order to minimise the risk of infection events, to limit them and to prevent the blanket closure of day-care centres and schools as a first measure [38], [39], [40]. Continuous attendance at a day-care centre [41] or school is not only essential for the sustainable educational success of the next generation, but also for healthy and successful development through social contacts, developmental tasks and challenges [42], [43], [44], [45]. In addition, it leads to a relief of the families and also to a release of the labour force of the persons having custody (compared to the continuous care of small children at home or the digital home-schooling of school children) [46], [47], [48], [49], [50], [51], [52].

In order to enable day-care centres and schools to operate on a regular basis as far as possible, in addition to the measures mentioned below it is necessary to develop a concept that allows [53], [54]

1. to distinguish respiratory infections caused by seasonal viral pathogens from those caused by SARS-CoV-2 using a risk-adapted testing strategy
2. to rapidly detect changes in the incidence of infection through a combination of tests based on specific risk factors (e.g. by taking into account holiday and travel risks, social history, membership of risk groups) and, if necessary, scientifically accompanied by sentinel examinations with involvement of carers and teachers;
3. to gain an overview of the developing epidemiological situation in small social niches (schools, day-care centres, districts) and to follow it regionally in order to be able to react in a timely and targeted (i.e. containment) manner;
4. making organisational arrangements to prevent the uncontrolled spread of infections within the facility. These concepts must be discussed in advance with the public health department. The possible contact persons in the event of an incident should be determined in advance. Parents, teachers, educators and carers should be informed in advance about the measures and their background;
5. to carry out structured outbreak investigations of clustered outbreaks in schools, day-care centres and municipalities in accordance with the general established principles of structured outbreak management by outbreak teams. The RKI has published guidelines for such a coordinated approach [55]. This guideline requires (as of 31.07.2020) a detailed update for the management of outbreaks in day-care centres and schools.

This concept must be regularly updated in the light of the continuing pandemic situation and adapted in line with new findings.

**The overall objective is to enable children and young people to attend day-care centres and schools in the future and to avoid a complete lockdown situation. At the same time, a safe working environment and adequate protection against infection should be prioritized for educators and teachers (and other adult contact persons involved in the facilities). This also applies to the families of the children, teachers and care personnel.**

## Maintenance of regular operations and prevention of SARS-CoV-2 out- breaks in day-care centres and schools

Almost without exception, the complete closure of day-care centers and schools as part of pandemic management was justified as a preventive measure. Reports of outbreaks of SARS-CoV-2 in day-care centres or schools include lists of a few cases of infection in which mostly teachers or other personnel were the source of infection[40], [56]. Less frequently, individual children or students whose parents were also often sick and were considered vectors [31], [57]. Children have so far not appeared as so-called “superspreaders” in schools and day-care centres or within families [29], [58], [59], [60], [61]. A more detailed analysis of the transmission paths in terms of a structured outbreak analysis is missing.

Most of the so-called outbreaks are accumulations of infections with smaller numbers of cases, the cause of which was outside the day-care centres or schools. There is no well-founded scientific evidence of an increased risk of transmission at day-care centres and schools, although it should be noted that the majority of the cluster events reported were carried out in a lockdown situation and many reports only refer to reports in local media [40]. **The re-opening of day-care centres and schools has not led to an increase in the number of infections among children and adolescents in any of the countries in which a general decline in infections in the general population has been observed and in which basic prevention measures in the general population have been further pursued (e.g. distance, cloth face masks, basic infection control rules).** Accordingly, the risk for teachers and care personnel to become infected through contacts in day-care and school operations – despite appropriate preventive measures – remained low. 

In countries in which outbreaks in schools were reported (e.g. Israel, Sweden or the USA), the number of infections in the general population was rising, in some cases significantly, so that the question of the causal relationships between infections in pupils through private or school transmissions and the cause of the infections in caregivers or staff remained unanswered [38], [40], [56]. Other aspects of specific risks in children and adolescents, such as the membership of families in population groups with religiously or ideologically motivated rejection of the hygiene measures [59], [62] or contradictory recommendations of the responsible health authorities with a simultaneously increased risk of mobility (USA) [63], [64] also make it difficult to draw general conclusions about the role of children and adolescents. The authors of a recent report on an outbreak at a secondary school in Israel emphasize that in the overcrowded school classes (>30 pupils), the wearing of mouth and nose covers and frequent airing were not consistently adhered to due to the high outside temperatures (40°C and more) (the air conditioning system was constantly in operation with no information on air exchange rates) [65].

Cases of SARS-CoV-2 infection also occur in community facilities or schools in Germany and other European countries. It can be assumed, however, that the incidence in day-care centres and schools is generally lower than the general incidence in the associated district.

Incidence data (Incidence here means the cumulative number [mean value] of new cases reported daily over the last 7 days per 100,000 inhabitants in the respective district; see Table 1 (Tab. 1)) apply to counties or municipalities. However, if a local outbreak can be controlled in a narrowly defined area, an increased incidence overall (in the district, the municipality) does not automatically mean that the prevention strategy in schools and day-care centres has to be adjusted (or that they have to be closed down completely). Through a timely and targeted adaptation (escalation) of preventive measures with separation of children and caregivers/teachers regarding location and time, ideally, when SARS-CoV-2 infections occur in day-care centres and schools, only the cluster directly affected (group/class, grade level) should be temporarily quarantined. However, this decision is the responsibility of the local health authority.

## Maintenance of regular operation with risk-based protection of staff in day-care centres and schools

The aim of the measures described below is to minimize the transmission of SARS-CoV-2 in day-care centres and schools. In everyday life, the prevention of any infection would be desirable, but probably cannot succeed and is therefore not the overall strategic goal. Such events cannot be completely ruled out, just as the entry of these infections into the facilities by the adults working there cannot be ruled out. The prevention of every single SARS-CoV-2 infection is not a realistic goal of a prevention strategy in which social life (and the age- and development-appropriate participation of children and adolescents) is maintained.

The staff’s anxieties resulting from this situation must be taken seriously. It requires specific measures that are the responsibility of the respective institutions. The lower risk of disease and transmission in children is accompanied by a lower risk of infection for teaching and care personnel if the basic hygiene rules described below are observed [25].

The risk of infection can be considerably minimized by a bundle of prevention measures. With regard to their implementation in day-care centres, explicit reference is made to the current version of the DAKJ’s Early Care and Child Health Commission [66], [67].

Teachers and educators are of paramount importance for the unrestricted access of children and adolescents to age-appropriate education and developmental opportunities within groups, classes and courses. Their daily work is not only relevant to the system but also to the future of our society [52], [68], [69]. The vast majority of the educational and teaching staff in day-care centres and schools are well aware of this, and they strive to fulfil their tasks with commitment even in these difficult and stressful times.

## Occupational health

Fears of infection among staff in day-care centres and schools should be addressed through professional education and advice. Fears should not lead to employees identifying as belonging to a “risk group” and withdrawing from their official duties without a definitive medical reason. This would expose children and adolescents and their families to the risk of considerable collateral damage [70], [71], [72]. An assessment by an occupational health physician [73] in consultation with the primary care physician is preferable to a self-assessment regarding the risk of increased vulnerability in the case of a SARS-CoV-2 infection. 

## Preventive measures of the staff

Based on the general (regional) and specific (within the institution) epidemiological situation, the following preventive measures must be taken by staff to protect themselves and others (prevention bundle) [74]:

- Observance of the infection control rules also in the private setting
  - Keeping distance whenever possible, especially to other adults and adolescents (i.e. communal areas for teachers or educators).
  - Washing hands, in special situations also hand disinfection.
  - Wearing a cloth face mask mask if distance rules cannot be observed [75].
- Regular airing at the end of each school hour (or hourly in day-care centres), if necessary with additional measurement of the CO_2_ content of the room air in special cases according to national recommendations (DGVU: https://dguv.de/corona-bildung/schulen/index.jsp); if necessary, critical assessment of rooms with air conditioning with regard to the proportion of fresh air or recirculated air (see footnote 4 in Table 1 (Tab. 1)).
- Early recognition of relevant symptoms in oneself, in colleagues and in the children and adolescents. The necessary consequences should be drawn promptly.
- Close communication (in strict compliance with the hygiene rules) among each other and cooperation of the institutional management with the local authorities.
- Active involvement of children and adolescents [76], parents (guardians) and their representatives in the overall concept of the respective institution. Parents are responsible for their children and indirectly also for the safe operation of the institution visited by their children (see below).

Before the complete closure of an institution [40], the escalation of preventive measures (see Table 1 (Tab. 1)) is an essential prerequisite for maintaining unrestricted or partially restricted regular operation.

## Outbreak prevention, management and measures

Due to the increasing evidence on the limited role of children (and probably also of adolescents up to 14 years of age) in the dynamics of the COVID-19 pandemic, the following advice should be taken into account and measures for day-care and school operation should be implemented:

1. An open, public discussion should lead to the acceptance of the fact that under pandemic conditions the elimination of any residual risk (the avoidance of every single SARS-CoV-2 infection without exception) is **not** possible in everyday life and therefore cannot be the overriding strategic goal. It is necessary to learn how to deal with SARS-CoV-2 infections and to minimize the risk of a local outbreak.
2. The normal operation of day-care centres and schools is possible in regions with a low incidence (Table 1 (Tab. 1)) of SARS-CoV-2 infections/COVID-19 cases while maintaining defined basic infection control standards.
3. Setting-specific conditions must receive special attention. Overall, the spectrum ranges from family-like structures in day-care to colleges with four-digit numbers of pupils.
4. Cancellation or restriction of regular operations requires a well-founded, structured and transparent political decision-making process, which focuses on safeguarding the educational, social and psychological interests of children and pupils while taking into account the risks of infection and illness. In particular, the interests of children and adolescents with special educational needs, special needs or supportive needs must be taken into account at least as much as those of other children and adolescents [42], [66], [67], [68], [74], [77]. This political process needs to be scientifically monitored. In most regions, it is possible to draw on the special expertise of paediatricians from various subdisciplines (including Paediatric Infectious Diseases) as well as infection control and environmental hygienists.
5. A complete closure of day-care centres or schools for reasons of preventive health protection or solely due to the accumulation of infections in the general population of a certain region without a definitive risk for the school or day-care centre is not justified.
6. Deviations from normal operation may become necessary due to an increasing incidence in a region or a defined outbreak. This planning should not happen at the time it becomes necessary but well in advance and these deviations must follow a step-by-step concept to be agreed upon with the responsible public health department. The complete closure of the entire facility should only be the last option if all other measured have failed.
7. When individual cases of infection occur in children, adolescents or persons of the care or teaching staff or in their immediate family or social environment, the primary goal is to limit the occurrence of infection to the defined area of a day-care group, a school class, or a course system. This is achieved by intensifying practised and previously established infection control rules, organising school operations according to constant group formation (cohorting) and prescribing defined quarantine measures or temporary closures of sub-areas.
8. In the higher class levels the fixed group formation may not be maintained due to requirements of course systems (i.e. no fixed classes or groups). On the other hand, these adolescents/young adults are quite capable of consistently implementing general infection prevention measures. Other forms of teaching can be offered here in addition to classroom teaching (e.g. digital learning).
9. Even in the event of infection clusters, **cluster isolation** should have priority over the closure of entire institutions. In cooperation with the responsible public health department, an infection control concept needs to be developed and agreed in advance, even before the facility is opened, to determine the procedures to be followed in individual cases of SARS-CoV-2 infections or outbreaks in an institution. A committee (outbreak and/or prevention team) of the school or day-care centre coordinated by the management of the facility is responsible for this.
10. When cases of infection occur, the ultimately responsible public health department, in cooperation with the institutional management, carries out a **structured outbreak management**. This involves an analysis of the transmission pathways according to established medical criteria (see RKI guidelines [55]) and the development of an adapted action plan for the specific case as well as testing the cohort regardless of symptoms to prevent subsequent asymptomatic transmission.
11. Complete day-care centre and school closures can only be a final step in the outbreak management if there has been an increase in transmission within the respective institution that cannot be contained by other measures.
12. While the institution management is an important partner, the local **health authority** is in charge. The involvement of representatives of educators, teachers, parents and children/adolescents should be part of the outbreak management.
13. The self-evaluation of teachers and other personnel as a risk group should be abandoned in favour of occupational health advice and assessment. The Committee for Occupational Medicine [78], considers the existence of defined pre-existing conditions without a defined age limit as a key criterion.
14. A general recommendation for teaching or care personnel to wear a daily mask during an outbreak should always be the most important step before a complete closure of a day-care centre or school. Irrespective of this, the rules of compulsory masks for older children and adolescents [75], [79], [80], [81], [82], [83], [84] as well as for teaching and care personnel in defined situations are part of preventive infection protection (see Table 1 (Tab. 1) and Table 2 (Tab. 2)).
15. The infection control concepts are based on the age of the pupils and the regional incidence of SARS-CoV-2 infections (see Table 1 (Tab. 1) and Table 2 (Tab. 2)). They are based on and supplement the institution-related hygiene plans of the respective federal states. An age limit of 10 years is considered reasonable, although this limit is blurred between a clearly low risk of transmission and illness in under 10-year-olds and an increasing risk behaviour and thus a growing transmission risk in the over-14-year-olds.
16. It is not possible for educators in day-care centres to adhere to distance rules and to wear a cloth face mask throughout the day when there is close contact with the children. Visors are no substitute for an MNB, but they can be used in different ways and should be preferred to not using a cloth face mask at all.

## Further measures

1. Establishment of easily accessible SARS-CoV-2 testing facilities for children, adolescents, caregivers and teachers, in order to enable rapid return to care facilities and workplaces without restrictions in the event of negative results.
2. Provide opportunities for testing on an ad hoc basis depending on aspects of special risks associated with travel, regional cluster events, special risks from family or social environment of children and pupils or other anamnestic evidence of special risks.
3. Symptom-independent sentinel SARS-CoV-2 studies in specific populations (in the context of scientific or scientifically supervised studies) to detect and prevent increased virus spread pre-emptively before manifest cases of disease occur.
4. Testing of sentinel populations (e.g. hospitalised children) with respiratory tract infections for respiratory viruses using multiplex PCR to map the general epidemiology of respiratory infectious diseases in a region (see also: https://influenza.rki.de/).
5. Ensuring vaccination for pupils and teachers or carers through active counselling and low-threshold vaccination programmes. As a result, other (vaccine-preventable) infections, which are often clinically indistinguishable from SARS-CoV-2 infections, become less frequent [85].

## Testing for SARS-CoV-2 in children in an outpatient treatment context

These instructions apply to children under 10 years of age. For older children and adolescents the relevant recommendations (https://www.rki.de/DE/Content/InfAZ/N/Neuartiges_Coronavirus/Massnahmen_Verdachtsfall_Infografik_DINA3.pdf?__blob=publicationFile) apply.

Sensitive methods for sample collection (e.g. saliva samples) [86] should be developed and validated as an alternative to the deep nasopharyngeal swab in children. Especially the deep nasal swab is very unpleasant for children and, depending on the age of the child, also traumatizing. In addition, the difficulties in obtaining material, especially for infants and toddlers, limit the validity of the results. In autumn and winter 2020, the usual significant (seasonal) increase in upper respiratory tract infections in children and adolescents can be expected. The seasonal occurrence of these infections can also be expected under the conditions of partial restriction of public life. 

**A distinction between a symptomatic SARS-CoV-2 infection and infections caused by other pathogens is not possible solely on the basis of clinical findings and examination.**

A general testing of all symptomatic children and adolescents for SARS-CoV-2 is unrealistic for logistical and capacity reasons [87]. In addition, if the prevalence in the investigated group is low, false-positive results could outweigh the correct positive results, with the consequence of additional retesting or expensive sequencing, or even lockdowns or quarantine measures, which undermine the confidence in hygiene management. 

The indication for a SARS-CoV-2 test should be individually justified based on the criteria listed below. Tests within the framework of scientific epidemiological studies or sentinel investigations as well as investigations within the framework of structured outbreak management should be evaluated separately. Early information of parents about this background is recommended.

**Sick children or adolescents in a reduced general condition** with fever, cough, sore throat or ear pain, severe abdominal pain, vomiting, diarrhoea or an unclear skin rash do not belong in either the day-care or school. Institutions are therefore entitled to have children or adolescents who have fallen ill being collected by their parents or guardians and to suggest a visit to a doctor.

**The request for a SARS-CoV-2 test is made by a physician (according to the criteria below) or by the health authorities. Institutions (schools, day-care centres etc.) are not entitled to demand a test (or the presentation of a negative test result).**

A medical certificate for readmission is only required if the child has been in quarantine due to COVID-19, a SARS-CoV-2 detection without symptoms or a Category 1 contact (according to RKI: https://www.rki.de/DE/Content/InfAZ/N/Neuartiges_Coronavirus/Kontaktperson/Management.html) with a SARS-CoV-2 positive person. Furthermore, institutions are not entitled to request a “negative test” as a condition for re-entry. Children with mild self-limiting signs of infection (mild cold without fever, only mild cough; these children mainly cough in the morning because the secretion runs down their throats at night) or after a short period of illness (less than 3 days) may be readmitted without a medical certificate if they are in good general condition and their symptoms have subsided. Some states have adopted the pragmatic solution whereby parents have to confirm in writing to the day-care centre that their child was free of symptoms for **24 hours** before being readmitted. For the coming year, it is recommended that such procedural arrangements be included in the care agreement between parents and facilities, thereby achieving commitment and certainty of action.

**A test for SARS-CoV-2 infection in an outpatient context should be carried out:**

- For children with **reduced general condition** and **symptoms of** infection, such as fever (sustained oral or rectal body temperature above 38°C or once above 38.5°C) or coughing for more than 2 days, children with gastrointestinal symptoms (persistent severe abdominal pain with or without diarrhoea and vomiting), **unless there is no other explanation** (as assessed by a physician).
- By order of the local health authorities (e.g. for tracing chains of infection) children living in the same household with a person who is infected with SARS-CoV-2 do not have to be necessarily tested, but remain in quarantine. This is decided by the public health department [87].

**Testing for SARS-CoV-2 infection in an outpatient context is not required in children in good general health with:**

- Rhinorrhoea (runny nose) or blocked nasal breathing with or without cough (without fever; yellowish or greenish secretion does not give a reliable indication as to whether the infection is bacterial or viral.)
- Mild self-limiting infection of less than 3 days (see above)
- clear diagnosis of a bacterial infection (e.g. tonsillopharyngitis caused by A-streptococci, urinary tract infection, skin and soft tissue infections) after clinical improvement and antibiotic therapy or other infection

## Note

The information on the test criteria corresponds to the status as of 03.08.2020 and may change depending on the epidemiological situation and new scientific findings. 

This statement was prepared and coordinated by the German Society for Pediatric Infectious Disease (DGPI), the German Society for Pediatric and Adolescent Medicine (DGKJ), the German Society for Hospital Hygiene (DGKH) and the German Academy for Children and Youth Medicine (DAKJ).

## Competing interests

This statement is supported by the German Society for Hygiene and Microbiology (DGHM), the Association of German Company and Works Doctors (VDBW e.V.) and the Society for Hygiene, Environmental Medicine and Preventive Medicine (GHUP).

The authors declare that they have no competing interests.

## Acknowledgement

The authors would like to thank Prof. Dr. Walter Haas and Prof. Dr. Wieler (Robert Koch Institute, Berlin) for their critical comments and further constructive suggestions (this is not an official statement of the RKI). The authors would also like to thank Prof. Dr. Dr. Schmidt-Chanasit of the Bernhard-Nocht-Institute, Hamburg, for the critical commentary and for the constructive comments on the virological basis of the statement.

## Figures and Tables

**Table 1:**
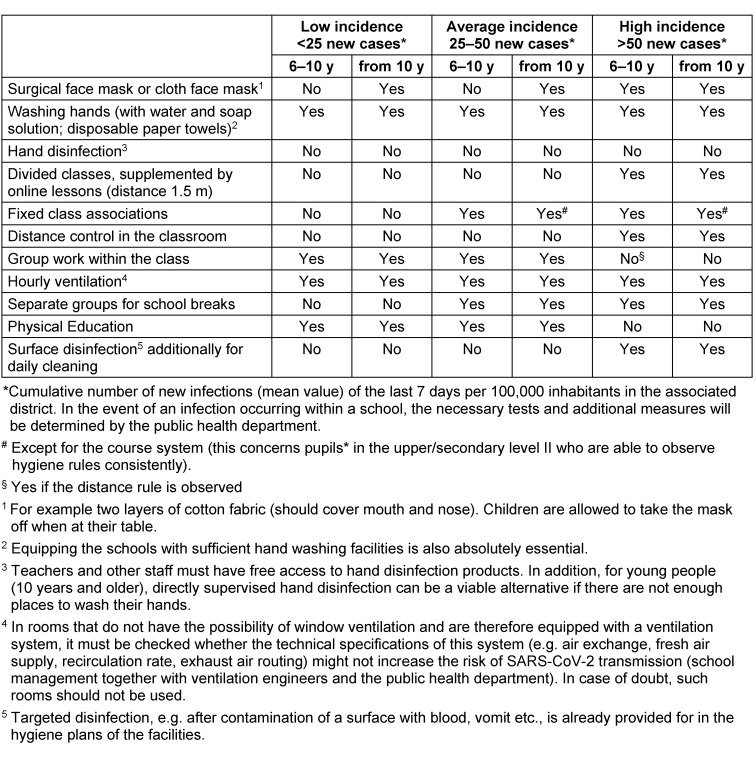
Measures for children and adolescents in primary and secondary schools

**Table 2:**
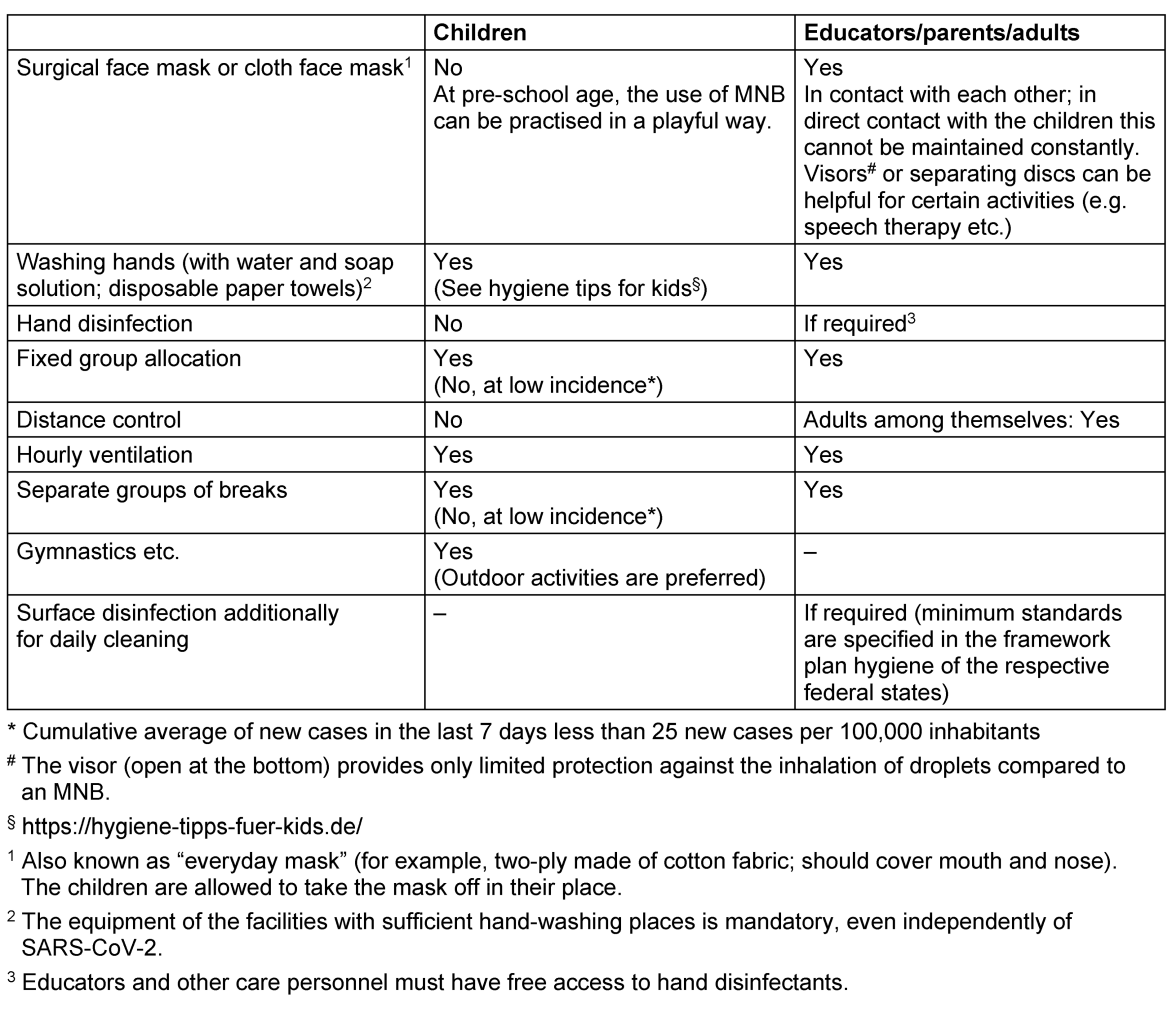
Measures in day-care centres and kindergarden
